# Autophagy regulates exosomal release of prions in neuronal cells

**DOI:** 10.1074/jbc.RA117.000713

**Published:** 2018-04-26

**Authors:** Basant A. Abdulrahman, Dalia H. Abdelaziz, Hermann M. Schatzl

**Affiliations:** From the ‡ Department of Comparative Biology & Experimental Medicine and; the § Calgary Prion Research Unit, University of Calgary, Calgary, Alberta T2N 4Z6, Canada,; the ¶ Department of Biochemistry and Molecular Biology, Faculty of Pharmacy, Helwan University, 11795 Cairo, Egypt, and; the ‖ Departments of Veterinary Sciences and of Molecular Biology, University of Wyoming, Laramie, Wyoming 82071

**Keywords:** prion, prion disease, exosome (vesicle), autophagy, CRISPR/Cas, Creutzfeldt–Jakob disease, extracellular vesicles, neurodegeneration, RT-QuIC, scrapie

## Abstract

Prions are protein-based infectious agents that autocatalytically convert the cellular prion protein PrP^C^ to its pathological isoform PrP^Sc^. Subsequent aggregation and accumulation of PrP^Sc^ in nervous tissues causes several invariably fatal neurodegenerative diseases in humans and animals. Prions can infect recipient cells when packaged into endosome-derived nanoparticles called exosomes, which are present in biological fluids such as blood, urine, and saliva. Autophagy is a basic cellular degradation and recycling machinery that also affects exosomal processing, but whether autophagy controls release of prions in exosomes is unclear. Our work investigated the effect of autophagy modulation on exosomal release of prions and how this interplay affects cellular prion infection. Exosomes isolated from cultured murine central neuronal cells (CAD5) and peripheral neuronal cells (N2a) contained prions as shown by immunoblotting for PrP^Sc^, prion-conversion activity, and cell culture infection. We observed that autophagy stimulation with the mTOR inhibitor rapamycin strongly inhibited exosomal prion release. In contrast, inhibition of autophagy by wortmannin or CRISPR/Cas9-mediated knockout of the autophagy protein Atg5 (autophagy-related 5) greatly increased the release of exosomes and exosome-associated prions. We also show that a difference in exosomal prion release between CAD5 and N2a cells is related to differences at the level of basal autophagy. Taken together, our results indicate that autophagy modulation can control lateral transfer of prions by interfering with their exosomal release. We describe a novel role of autophagy in the prion life cycle, an understanding that may provide useful targets for containing prion diseases.

## Introduction

Prion diseases are fatal, infectious neurodegenerative disorders of man and animals that can be transmitted within and sometimes between species (1, 2). Examples are Creutzfeldt–Jakob disease, kuru and variant Creutzfeldt–Jakob disease in humans, scrapie in sheep and goat, bovine spongiform encephalopathy in cattle and other species, and chronic wasting disease in deer, elk, and moose (3–6). Loss of neurons is a key feature of prion diseases, accompanied by astrogliosis and mild microglia activation. This results in a progressive spongiform degeneration of the CNS,^3^ leading to ataxia, behavioral changes and, in humans, highly progressive loss of intellectual abilities (2, 7–9). The formation of the abnormally folded, protease-resistant isoform PrP^Sc^ of the host-encoded cellular prion protein (PrP^C^) is the underlying mechanism in prion disease pathogenesis (1, 2). PrP^Sc^ is derived post-translationally from PrP^C^ by a profound conformational change (10, 11). The exact physiological function of PrP^C^ is unclear (7), although roles in copper transport and neuroprotection are evident among the many potential roles attributed (12–14). PrP^C^ was also described as receptor for oligomeric β-amyloid (15). Recently, a role of PrP^C^ in regulating exosomal release was reported (16).

In prion-infected cultured neurons and in neurons of brain samples from prion disease affected humans, the appearance of multivesicular bodies (MVBs) and autophagic vacuoles has been reported (17–19). Interestingly, MVBs are known to give rise to exosomes. Exosomes are nanovesicles secreted by most cell types (20–23). They are formed intracellularly by inward budding of the limiting membrane of endocytic compartments. MVBs ultimately fuse with the plasma membrane, releasing their internal vesicles into the extracellular milieu (23). Exosomes are secreted into various body fluids such as plasma, urine, milk, amniotic fluid, and saliva, making them interesting candidates for novel biomarkers (24, 25). Exosomes are secreted by most cells in a constitutive manner and play a role in cell-to-cell communication (26–28).

It has been shown that exosomes can contain prions (29–31) and that they participate in delivering PrP^Sc^ to uninfected neighboring cells (32–34). We reported previously that cell-free cell culture media of prion-infected hypothalamic ScGT1 cells can induce PrP^Sc^ formation in recipient cells, indicating a cell-free transfer of prion infection (18). Exosomes also play a role in spreading the toxic forms of other aggregated proteins such as α-synuclein, β-amyloid, and misfolded SOD1 (35–38). A pivotal role of autophagy in the secretion of exosomes is emerging (39, 40). In macroautophagy (here referred to as autophagy), autophagosomes engulf cytosolic macromolecules and deliver them to lysosomes for degradation (41–43). The clearance of aggregation-prone proteins, such as mutant huntingtin fragments or mutant forms of α-synuclein causing Huntington's and Parkinson's disease, respectively, can be mediated by autophagy (44, 45). Animal models of Huntington's disease and of other proteinopathies revealed that treatment with rapamycin, a known inducer of autophagy, accelerates the clearance of toxic proteins (46–49). Of note, changes in autophagy level affect MVB formation and exosomal release. Upon stimulating autophagy, MVBs fuse more with autophagic vacuoles, resulting in inhibition of exosomal release (50, 51). The beneficial effect of up-regulated autophagy in prion disease models *in vitro* and *in vivo* has been described by us and others (52–58).

The impact of autophagy on exosomal release of prions is not known. In this work, we address the link between autophagy, exosomes and release of prion infectivity in two different cell culture models. Persistently prion-infected ScCAD5 cells are used as neuronal cells of CNS origin; ScN2a cells represent peripheral nervous system neuronal cells. Upon stimulating autophagy using rapamycin, we found a decrease in exosomal release and exosome-associated prions. On the other hand, disruption of the autophagic machinery by knocking out Atg5 using CRISPR/Cas9 technology resulted in increased release of exosomes and increase in exosome-associated PrP^Sc^. Similarly, inhibition of autophagy using wortmannin (phosphatidylinositol 3-kinase inhibitor) enhanced the secretion of exosomes and of exosomal PrP^Sc^. In summary, our work shows that autophagy modulation controls exosomal release and, consequently, secretion of prions in exosomes. These findings help to better understand how autophagy can control cellular prion infection, not only via regulation of autophagosomal-lysosomal prion clearance but also by controlling exosomal release of prions.

## Results

### Isolation and characterization of exosomes from prion infected ScCAD5 and ScN2a cells

**Figure 1.**
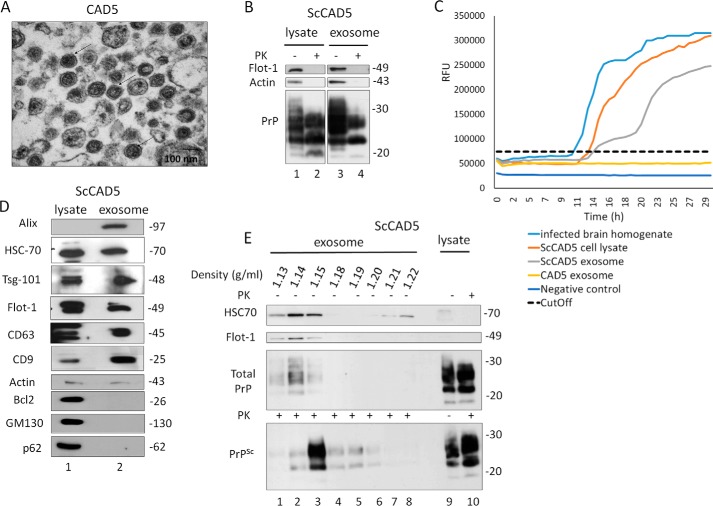
**Characterization of exosomes isolated from CAD5/ScCAD5 neuronal cells.** *A*, representative TEM of exosomes isolated from CAD5 culture medium reveals a homogenous population of vesicles of 100 nm in diameter characteristic for exosomes (some denoted by *black arrows*). *Scale bar*, 100 nm. *B*, immunoblot of ScCAD5 cell lysate and exosome preparations probed for total PrP (−PK) and PrP^Sc^ (+PK) (anti-PrP mAb 4H11). Actin was used as loading control. Flotillin-1 (*Flot-1*) was used as an exosome marker. *C*, RT-QuIC of CAD5 exosome, ScCAD5 exosome, ScCAD5 cell lysate, 10% brain homogenate from terminally prion-sick mice (22L) or left unseeded (negative control). The average increase of thioflavin-T fluorescence of replicate wells is plotted as a function of time. The *y* axis represents RFU, and the *x* axis represents time (h). *D*, immunoblot analysis of ScCAD5 cell lysate and exosomes isolated from ScCAD5 culture medium. Exosome preparation is positive for exosome markers Alix, HSC70, Tsg-101, flotillin-1, CD63, and CD9 and negative for mitochondrial marker Bcl2, Golgi marker GM130, and nuclear marker nucleoporin p62. Actin was used loading control. *E*, ScCAD5 exosome pellet loaded on the top of a continuous sucrose gradient and ultracentrifuged. Fractions were analyzed by Western blotting and probed for HSC70, flotillin-1, and mAb 4H11 to detect PrP/PrP^Sc^. *Lanes 9* and *10* represent cell lysate before and after PK digestion, respectively.

**Figure 2.**
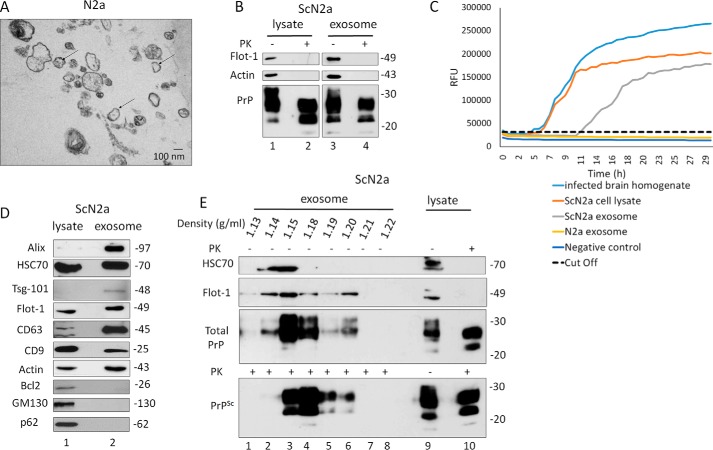
**Characterization of exosomes isolated from N2a/ScN2a cells.** *A*, representative TEM of exosomes isolated from N2a culture medium shows a population of vesicles of 100 nm in diameter (some denoted by *black arrows*). *Scale bar*, 100 nm. *B*, immunoblot of ScN2a cell lysate and exosome preparations probed for total PrP (−PK) and PrP^Sc^ (+PK) (anti-PrP mAb 4H11). Flotillin-1 (*Flot-1*) was used as exosome marker. Actin was used as loading control. *C*, RT-QuIC of N2a exosome, ScN2a exosome, ScN2a cell lysate, and 10% brain homogenate from terminally prion-sick mice (22L) or left unseeded (negative control). The average increase of thioflavin-T fluorescence of replicate wells is plotted as a function of time. The *y* axis represents RFU, and the *x* axis represents time in hours. *D*, immunoblot analysis of ScN2a cell lysate and exosomes isolated from ScN2a cell culture medium. Exosome preparation is positive for exosome markers Alix, HSC70, Tsg-101, flotillin-1, CD63, and CD9 and negative for mitochondrial marker Bcl2, Golgi marker GM130, and nuclear marker nucleoporin p62. Actin was used as loading control. *E*, ScN2a exosome pallet loaded on the top of a continuous sucrose gradient and ultracentrifuged. The fractions were analyzed by Western blotting and probed for HSC70, flotillin-1, and mAb 4H11 to detect total PrP and PrP^Sc^. *Lanes 9* and *10* are cell lysate before and after PK digestion, respectively.

To study the effect of autophagy on exosomal release of prions, we used two prion-infected cell culture models. The murine cell lines ScCAD5 (CNS, catecholaminergic/neuronal) and ScN2a (peripheral nervous system, neuroblastoma/neuronal) are both persistently infected with the mouse-adapted scrapie prion strain 22L (59, 60). Exosomes isolated from cell culture media of ScCAD5 and ScN2a cells were characterized as shown in Figs. 1 and 2, respectively. The protein markers for exosomes flotillin-1, Alix, Tsg101, CD63, CD9, and HSC70 were detected in isolated exosomes from both cell lines using immunoblotting (Figs. 1*D* and 2*D*). The purity of isolated exosomes was confirmed by lack of Golgi (GM130), mitochondrial (Bcl2), and nuclear (nucleoporin p62) contaminants (Figs. 1*D* and 2*D*). Transmission EM (TEM) imaging of exosomal preparations showed vesicles with approximate diameters from 80 to 150 nm, and the morphology of vesicles was consistent with the previously reported cup shape of exosomes (Figs. 1*A* and 2*A*) (61, 62). To address in these cell models the role of exosomes in the release of prion proteins (both cellular and pathological isoforms) into the extracellular milieu, exosomes isolated from cell-free culture media of ScCAD5 and ScN2a cells were subjected to proteinase K digestion, and then both nontreated (−PK) and treated (+PK) fractions were analyzed in immunoblot for PrP using the monoclonal anti-PrP antibody 4H11. As expected, total PrP (−PK) and PrP^Sc^ (+PK) were detected in exosomes, which confirms the packaging of both PrP isoforms into exosomes in these cell lines **(**Figs. 1*B* and 2*B*). For further characterization of the exosomes, we analyzed the fractionation of 100,000 × *g* exosome isolate in a continuous sucrose gradient. As expected, the exosomes reside in fractions with density ranged from 1.13 to 1.18 g/ml (Figs. 1*E* and 2*E*). This fraction's density range has been previously reported to contain the exosomes, which indicates the high purity of our exosome isolates (23, 28). Interestingly, both total PrP and PrP^Sc^ were enriched in the same density gradient fractions as the exosomes, indicating that secreted PrP^Sc^ may be associated with the exosomes in both cell lines (Figs. 1*E* and 2*E*). Of note, some PrP^Sc^ was found in the collected pellets at all steps of the differential centrifugation (2,000 × *g* and 10,000 × *g*), indicating a considerable amount of PrP^Sc^ released into the culture media in both cell lines (Fig. S1, *A* and *B*).

There is mounting evidence that exosomes containing PrP^Sc^ are able to disseminate prions from cell to cell (29, 30). Notably, the exosomes isolated from the ScCAD5 cells could successfully infect naïve CAD5 cells as shown by Fig. S3. Even though partial resistance of PrP to PK digestion is a main determinant for prion infectivity, several reports have demonstrated that prion infectivity is not always related to PK resistance. This led us to test the infectivity of PrP packaged into exosomes using real-time quaking-induced conversion (RT-QuIC) assay. RT-QuIC is a highly sensitive *in vitro* amplification technique used for detection of prions and diagnosing prion diseases in various tissues and body fluids (63, 64). Prion infectivity of exosomes was assessed previously using prion-infected cell assay (34, 61) and protein misfolding cyclic amplification; however, detection of prion infectivity in exosomes using RT-QuIC is lacking. Exosomes isolated from ScCAD5 and ScN2a cells revealed significant conversion of recombinant mouse PrP into ThT-binding aggregates in RT-QuIC compared with exosomes from noninfected cells, indicating the presence of appreciable amounts of prion-seeding activity (Figs. 1*C* and 2*C*). In ScCAD5 cells, conversion started after 12 h (lag phase) and reached maximum relative florescence units (RFU) comparable to the corresponding cell lysate and brain homogenate of terminally prion-sick mice (used as positive control) (Fig. 1*C*). Exosomes isolated from ScN2a cells showed comparable prion-conversion activity, starting after 11 h (lag time) with maximum florescence equivalent to conversion induced by the cell lysate (Fig. 2*C*). Taken together, exosomes isolated from both cell lines contained PrP^Sc^ and were positive for prion-conversion activity, indicating release of *bona fide* prion infectivity.

### Induction of autophagy reduces exosomal release of prions in ScCAD5 and ScN2a cells

Having established that both of our cell models released prions in exosomes, we wanted to study how manipulation of autophagy affects exosomal release of prions. Pharmacological induction of autophagy was reported to have anti-prion effects *in vitro* and *in vivo* by promoting the intracellular degradation of PrP^Sc^ in lysosomes (53, 54). There is growing evidence that the autophagy machinery and exosomal release are interconnected (65, 66). However, the cross-talk between autophagy and exosomes in modulating the spread of prion infection between cells is still elusive.

**Figure 3.**
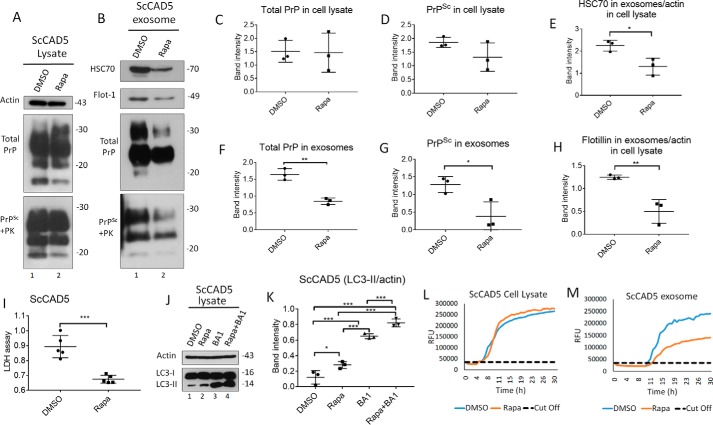
**Autophagy stimulation decreases exosome secretion and consequently impacts exosomal PrP^Sc^ in ScCAD5 neuronal cells.** *A* and *B*, Western blotting of cell lysate and exosomes from ScCAD5 cells treated with 500 nm of rapamycin (*Rapa*) or solvent only (DMSO). HSC70 and flotillin-1 (*Flot-1*) were used as exosome markers. PrP (−/+ PK) was probed with mAb 4H11. *C* and *D*, densitometric analysis for either total PrP or PrP^Sc^, respectively, from ScCAD5 cell lysate normalized with actin (± S.D.) after treatment with 500 nm rapamycin or DMSO (*n* = 3 experiments). *E* and *H*, densitometric analysis for exosomal HSC70 and flotillin-1, respectively, normalized with actin in the corresponding cell lysate (± S.D.) after treatment with rapamycin or DMSO (*n* = 3 experiments). *, *p* < 0.05; **, *p* < 0.01. *F* and *G*, densitometric analysis for either total PrP or PrP^Sc^, respectively, from ScCAD5 exosomes normalized with actin in the corresponding cell lysate (± S.D.) after treatment with rapamycin or DMSO (*n* = 3 experiments). **, *p* < 0.01. *, *p* < 0.05. *I*, lactate dehydrogenase (*LDH*) cytotoxicity assay (OD = 490 nm). Supernatant from ScCAD cells treated with rapamycin or DMSO for 6 days was tested to detect the level of LDH (± S.D.; *n* = 5 replicates). ***, *p* < 0.001. *J*, Western blotting of cell lysate of ScCAD5 cells treated with vehicle only (DMSO), 500 nm of rapamycin, 100 nm of bafilomycin A1 (*BA1*), or rapamycin + bafilomycin A1 for 4 h. LC3 was used to measure the autophagic flux, and actin was used as loading control. *K*, densitometric analysis for LC3-II protein levels normalized with actin (± S.D.; *n* = 3 replicates). *, *p* < 0.05; ***, *p* < 0.001. *L* and *M*, RT-QuIC for either ScCAD5 cell lysate or exosomes, respectively. The cells were treated with rapamycin or solvent only (DMSO).

**Figure 4.**
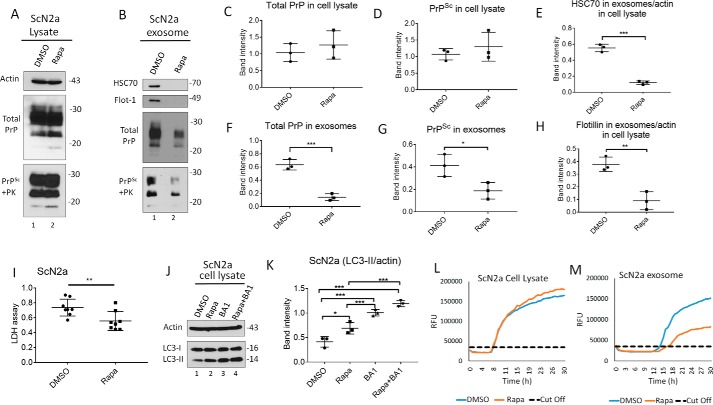
**Autophagy stimulation mitigates exosome secretion and significantly decreases exosomal PrP^Sc^ in ScN2a cells.** *A* and *B*, Western blotting of cell lysate and exosomes from ScN2a cells treated with 500 nm of rapamycin (*Rapa*) or solvent only (DMSO) for 6 days. HSC70 and flotillin-1 (*Flot-1*) were used as exosome markers. PrP (−/+ PK) was probed with mAb 4H11. Actin was used as a loading control for cell lysate. *C* and *D*, densitometric analysis for either total PrP or PrP^Sc^, respectively, from ScN2a cell lysate normalized with actin (± S.D.) after treatment with rapamycin or DMSO (*n* = 3 experiments). *E* and *H*, densitometric analysis for exosomal HSC70 and flotillin-1, respectively, normalized with actin in the corresponding cell lysate (± S.D.; *n* = 3 experiments). ***, *p* < 0.001; **, *p* < 0.01. *F* and *G*, densitometric analysis for either total PrP or PrP^Sc^, respectively, from ScN2a exosomes normalized with actin in the corresponding cell lysate (± S.D.; *n* = 3 experiments). ***, *p* < 0.001; *, *p* < 0.05. *I*, lactate dehydrogenase (*LDH*) cytotoxicity assay (OD = 490 nm). Supernatant from ScN2a cells treated with 500 nm rapamycin or DMSO for 6 days was tested to detect the level of LDH (± S.D.; *n* = 8 replicates). **, *p* < 0.01. *J*, Western blotting of cell lysate of ScN2a cells treated with vehicle only (DMSO), 500 nm of rapamycin, 100 nm of bafilomycin A1 (*BA1*), or rapamycin + bafilomycin A1 for 4 h. LC3 was used to measure the autophagic flux, and actin was used as loading control. *K*, densitometric analysis for LC3-II protein levels normalized with actin (± S.D.; *n* = 3 replicates). *, *p* < 0.05; ***, *p* < 0.001. *L* and *M*, RT-QuIC for either ScN2a cell lysate or exosomes; respectively; the cells were treated with rapamycin or solvent only (DMSO).

To study the role of autophagy induction in modulating secretion of exosomes and release of prions, we treated ScCAD5 and ScN2a cells with rapamycin, which is an mTOR-dependent autophagy stimulator. Rapamycin was first tested for potential toxicity in CAD5 and N2a cells. We found no significant toxicity for the concentrations used in this study when compared with vehicle-treated cells, yet the LDH release of rapamycin-treated cells was significantly lower than that of control cells, suggesting better survival with the rapamycin treatment (Figs. 3*I* and 4*I*). Additionally, rapamycin was confirmed to stimulate autophagy in both cell lines as shown by increase in LC3II signal (Figs. 3, *J* and *K*, and 4, *J* and *K*). In both cell lines, rapamycin treatment caused a significant decrease in exosome release (Figs. 3, *B*, *E*, and *H*, and 4, *B*, *E*, and *H*) and exosomal total PrP and PrP^Sc^ contents (Figs. 3, *B*, *F*, and *G*, and 4, *B*, *F*, and *G*). However, no change in total PrP and PrP^Sc^ amounts was found in the cell lysates (Figs. 3, *A*, *C*, and *D*, and 4, *A*, *C*, and *D*). Interestingly, the reduction in exosomal release caused by rapamycin treatment was as pronounced as that induced by GW 4869, which is widely used as an inhibitor for exosomal release (Fig. S1, *C* and *D*) (62). Next, we wanted to correlate observed effects on exosomal PrP^Sc^ to prion infectivity by testing prion-conversion activity of exosomes using RT-QuIC. Exosomes isolated from rapamycin-treated ScCAD5 and ScN2a cells showed less conversion activity compared with vehicle-treated controls for the same dilution of seed (Figs. 3, *L* and *M*, and 4, *L* and *M*). Taken together, our results demonstrate that induction of autophagy in prion-infected neuronal cells reduces the release of exosomes, paralleled by a marked reduction of prion infectivity in exosomes.

### Inhibition of autophagy increases exosomal release of prions in ScCAD5 and ScN2a cells

**Figure 5.**
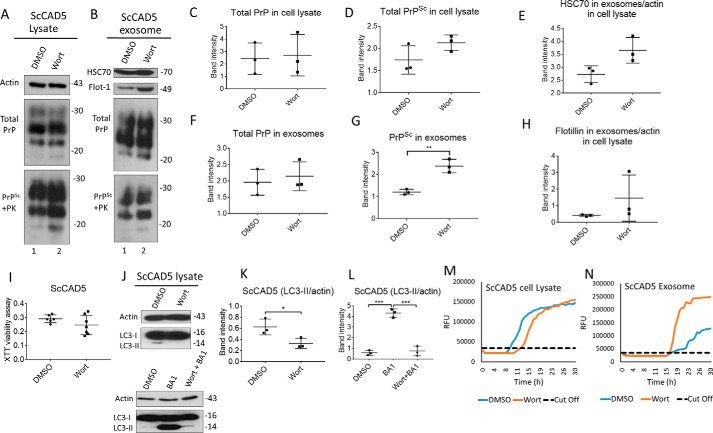
**Inhibition of autophagy increases exosome release and exosomal PrP^Sc^ in ScCAD5 cells.** *A* and *B*, Western blotting of cell lysate and exosomes from ScCAD5 cells, respectively, treated with 4 nm of wortmannin (*Wort*) for 48 h or solvent only treated (DMSO). HSC70 and flotillin-1 (*Flot-1*) were used as exosome markers. Actin was used as loading control for cell lysate. PrP (−/+ PK) was probed with mAb 4H11. *C* and *D*, densitometric analysis for either total PrP or PrP^Sc^, respectively, from ScCAD5 cell lysate normalized with actin (± S.D.) after treatment with 4 nm of wortmannin or DMSO (*n* = 3 experiments). *E* and *H*, densitometric analysis for exosomal HSC70 and flotillin-1, respectively, normalized with actin in the corresponding cell lysate (± S.D.; *n* = 3 experiments). *F* and *G*, densitometric analysis for either total PrP or PrP^Sc^, respectively, from ScCAD5 exosomes normalized with actin in the corresponding cell lysate (± S.D.; *n* = 3 experiments). **, *p* < 0.01. *I*, XTT viability assay. ScCAD5 cells were treated with 4 nm wortmannin or DMSO for 48 h; then cell viability was detected based on metabolic activity (± S.D.; *n* = 7 replicates). *J*, *upper panel*, Western blotting of cell lysate of ScCAD5 cells treated with vehicle only (DMSO) or 4 nm of wortmannin for 4 h. *Lower panel*, Western blotting of cell lysate of ScCAD5 cells treated with vehicle only (DMSO), 4 nm of wortmannin, or wortmannin with bafilomycin A1 (*Wort* + *BA1*) for 4 h. LC3 was used to measure the autophagic flux and actin was used as loading control. *K* and *L*, densitometric analysis for LC3-II protein levels normalized with actin (± S.D.; *n* = 3 replicates). *, *p* < 0.05; ***, *p* < 0.001. *M* and *N*, RT-QuIC for either ScCAD5 cell lysate or exosomes, respectively. The cells were treated with wortmannin or solvent only (DMSO).

Given that increased autophagy reduces release of prions in exosomes, we next wanted to study whether a compromised autophagy would have opposite effects. To do so, autophagy was suppressed in ScCAD5 using wortmannin, a well known autophagy suppressor, which inhibits class III PI3K (67). The wortmannin concentration used in our study (4 nm) was nontoxic for CAD5 cells as shown by XTT assay (Fig. 5*I*). Although the basal autophagy in CAD5 cells is not very prominent, wortmannin was able to abolish the signal of LC3II in CAD5 cells indicating a successful inhibition of autophagy (Fig. 5, *J–L*). Interestingly, ScCAD5 cells with such compromised autophagy exhibited a slight increase in exosomes released (Fig. 5, *B*, *E*, and *H*), yet this increase in exosomal release was not statistically significant. Importantly, this was associated with significantly increased PrP^Sc^ amount in the exosomes (Fig. 5, *B*, *F*, and *G*). Additionally, wortmannin increased the infectivity of the ScCAD5 released exosomes as shown by RT-QuIC (Fig. 5, *M* and *N*). The effect of compromised autophagy on exosomal release was similar to the effects of treatment with monensin, a drug reported to stimulate the packaging of PrP^Sc^ into exosomes and the intercellular transfer of prion infectivity (34) (Fig. S1*E*).

Next, we wanted to genetically compromise autophagy in ScCAD5 cells. The key autophagy protein Atg5 was knocked down using siRNA (siAtg5) (Fig. S2). We only achieved an incomplete transient knockdown of Atg5. Consequently, ScCAD5 cells with such compromised autophagy exhibited a minor increase in exosomes released (Fig. S2).

**Figure 6.**
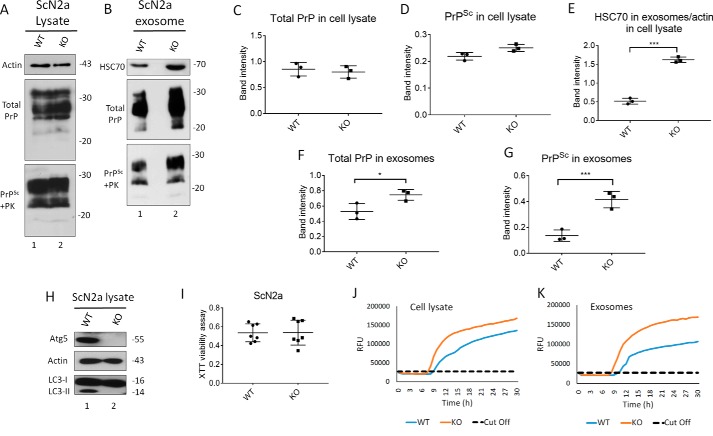
**Knockout of Atg5 increases exosome release and exosomal PrP^Sc^ in ScN2a cells.** *A* and *B*, Western blotting of cell lysate and exosomes from ScN2a cells respectively, either WT or KO for Atg5. HSC70 was used as exosomal markers. Total PrP and PrP^Sc^ were detected using mAb 4H11 antibody. *C* and *D*, densitometric analysis for either total PrP or PrP^Sc^, respectively, from WT or KO ScN2a cell lysate normalized with actin (± S.D.; *n* = 3 experiments). *E*, densitometric analysis for exosomal HSC70 normalized with actin in the corresponding cell lysate (± S.D.; *n* = 3 experiments). ***, *p* < 0.001. *F* and *G*, densitometric analysis for either total PrP or PrP^Sc^, respectively, from WT or KO ScN2a exosomes normalized with actin in the corresponding cell lysate (± S.D.; *n* = 3 experiments). ***, *p* < 0.001; *, *p* < 0.05. *H*, immunoblot showing complete knockout of Atg5 compared with WT ScN2a cells. Actin was used as loading control. Atg5 KO resulted in complete absence of LC3-II, confirming disruption of autophagy machinery. *I*, XTT viability assay. (OD = 490 nm). The cells were cultured for 48 h for XTT viability assay (*n* = 7 replicates). *J* and *K*, RT-QuIC for WT or KO ScN2a cells.

Using CRISPR/Cas9 technology, we generated Atg5-deficient ScN2a cells (KO), which are defective in autophagy (Fig. 6*H*). Notably, the Atg5-null ScN2a cells showed normal viability compared with their WT counterparts (Fig. 6*I*). Atg5-KO ScN2a cells revealed significantly higher exosome release compared with WT control cells, as shown by HSC70 signals in immunoblots (Fig. 6, *B* and *E*). Importantly, this was associated with strongly increased total PrP and PrP^Sc^ amounts in exosomes (Fig. 6, *F* and G, respectively). However, no change occurs in total PrP and PrP^Sc^ levels in the cell lysates (Fig. 6, *A*, *C*, and *D*). Testing prion-conversion activity by RT-QuIC demonstrated shorter lag time and higher maximum florescence (RFU) for exosomes from Atg5-KO cells, indicating higher prion-seeding activity compared with exosomes isolated from WT ScN2a cells (Fig. 6*K*). Notably, the effect of autophagy blockage on the induction of the exosome release in ScN2a-KO cells was comparable with the effect of treating ScN2a cells with monensin, which has been reported to increase exosomal release from neuronal cells (Fig. S1*F*). Taken together, blocking autophagy, either partially or completely, boosts release of prions in exosomes and consequently increases intercellular spread of prion infection.

### ScCAD5 cells release more prions in exosomes and have a lower level of autophagy than ScN2a cells

**Figure 7.**
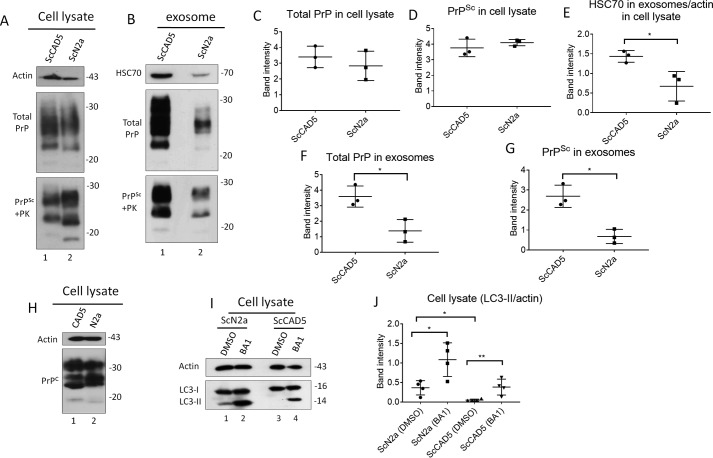
**ScCAD5 cells release more exosomes and PrP^Sc^ compared with ScN2a cells, which is inversely correlated to autophagy competence.** Comparable numbers of ScN2a and ScCAD5 cells were used in this experiment. *A* and *B*, cell lysate and exosomes isolated from conditioned media from ScCAD5 and ScN2a cells were analyzed in immunoblot for HSC70 and PrP. *C* and *D*, densitometric analysis for either total PrP or PrP^Sc^ respectively from ScCAD5 or ScN2a cell lysate normalized with actin (± S.D.; *n* = 3 experiments). *E*, densitometric analysis for exosomal HSC70 normalized with actin in the corresponding cell lysate (± S.D.; *n* = 3 experiments). *, *p* < 0.05. *F* and *G*, densitometric analysis for either total PrP or PrP^Sc^, respectively, from ScCAD5 or ScN2a exosomes normalized with actin in the corresponding cell lysate (± S.D.; *n* = 3 experiments). *, *p* < 0.05. *H*, immunoblot comparing PrP^C^ levels between CAD5 and N2a cells using mAb 4H11. *I*, immunoblot comparing the level of LC3-I to LC3-II between ScN2a and ScCAD5 cells with and without treatment with bafilomycin A1 (*BA1*) for 4 h. Actin was used as a loading control. *J*, densitometric analysis for LC3-II protein levels normalized with actin (± S.D.; *n* = 4 replicates). *, *p* < 0.05, **, *p* < 0.01.

To explore the difference between CAD5 and N2a neuronal cells in terms of autophagy competence and exosomal release, we isolated exosomes from the same number of ScCAD5 and ScN2a cells. We found that the exosome yield obtained from ScCAD5 cells was markedly higher than that isolated from almost same number of ScN2a cells, as shown by HSC70 signal in immunoblot analysis (Fig. 7, *B* and *E*). Although ScCAD5 and ScN2a cells have comparable amounts of PrP^total^ in the lysate, the PrP levels in ScCAD5 isolated exosomes are significantly higher than ScN2a exosomes (Fig. 7, *A–D*, *F*, and *G*). Then we wanted to correlate this to the level of basal autophagy in both cell types. Basal autophagic flux, as measured by LC3-II signals, was negligible in ScCAD5 cells compared with that in ScN2a cells (Fig. 7, *I* and *J*). A recent study reported that the level of PrP^C^ plays a role in controlling exosomal release (16). This led us to test the difference in PrP^C^ between uninfected CAD5 and N2a cells. We found that PrP^C^ levels in CAD5 and N2a cells are not very different (Fig. 7*H*).

In summary, the lower level of basal autophagy could be an explanation for the higher exosomal release of prions from ScCAD5 cells. This may facilitate spreading of prions to neighboring cells and explain their high susceptibility to prion infection.

## Discussion

Exosomes are nanovesicles secreted by almost all cell types into the extracellular space, which contains proteins, lipids, and nucleic acids that are derived from the parent cell. They play a crucial role in intercellular communication, both locally and systemically. This includes immune regulation, tissue repair, and neuronal and neuron–glia communication (39, 68–70). Exosomes originate from MVBs, which are late endosomes and hence a part of the endosomal system (23). Whereas exosomes are constitutively released from most cells under normal physiological conditions, their formation and release can be modulated by infection and cellular stress (71). The presence of exosomes with their cargo in body fluids makes them optimal platforms for intercellular transmission of diseases. This includes neurodegenerative disorders which involve prion and prion-like mechanisms (30, 72–75).

### High prion-conversion activity in exosomes

Here, we use prion-infected cells to study the exosomal release of prions and the cellular programs involved. Notably, we detected significant prion-conversion activity in exosomes isolated from culture media of ScCAD5 and ScN2a cells using RT-QuIC, which is one of the most sensitive and specific techniques for detection of prion-seeding activity (63). Interestingly, infectivity in exosomes isolated from ScCAD5 cells was comparable with that of brain homogenates from mice with terminal prion disease and the cell lysates of ScCAD5 cells in terms of RT-QuIC curve kinetics. Considering that exosomes are highly diluted compared with prions as found in brain homogenate and cell lysate, the high prion-seeding activity of exosomes suggests that prion infectivity is very efficiently packaged into such extracellular vesicles. Interestingly, the exosomes isolated from the ScCAD5 cells efficiently infect naïve CAD5 cells (Fig. S3). This underscores the potential impact of exosomes in spreading prion infectivity to neighboring and more distant cells in the brain, in accordance with several previous reports (28, 30, 61).

The molecular and cellular mechanisms that influence the packaging and release of prions into exosomes are not fully understood. A recent study has demonstrated a role for the neutral sphingomyelinase pathway in packaging PrP^Sc^ into exosomes, albeit it did not exclude the involvement of other controlling pathways (62). In the current study, we demonstrate that autophagy, a basic cellular housekeeping and degradation machinery, controls the release of prions into exosomes.

### Enhanced autophagy decreases exosomal release of prions

We show here that pharmacological induction of autophagy by rapamycin remarkably inhibits release of exosomes in ScCAD5 cells, a central nervous system cell line, and ScN2a cells, which are derived from the peripheral nervous system. This is paralleled by reduction of prion-conversion activity and PrP^Sc^ in such exosomes. Intriguingly, limiting the exosome release and hence the intercellular prion dissemination could be one of the ways used by autophagy to control the prion infection. At the cellular level, the effect of autophagy stimulation on exosome release can be explained by modulating the trafficking of MVBs. The latter fuse more with autophagosomes, the typical degradative and recycling organelles. This is in agreement with reports showing that induction of autophagy by starvation or LC3 overexpression enhances the fusion of MVBs with autophagic vacuoles (50, 51). There is a growing body of evidence showing that autophagy, MVBs, and exosome release are tightly interconnected (39). In addition to removal of harmful or unwanted material in exosomes in cellular stress situations, this mechanism has a general role in organizing unconventional secretion of proteins that are devoid of a signal peptide (40, 76–78). Despite efforts toward understanding the packaging of proteins and protein aggregates into exosomes, the factors that modulate whether fusion of MVBs with autophagic vacuoles results in release of exosomes or degradation in lysosomes are not fully elucidated.

### Dysfunctional autophagy increases release of prions in exosomes

On the other side, inhibition of autophagy either by wortmannin in ScCAD5 cells or Atg5 knockout in ScN2a cells markedly promotes release of exosomes. Not unexpected, this was accompanied by release of prion infectivity in exosomes as revealed by our RT-QuIC results. This indicates that a dysfunctional autophagy may contribute to lateral spreading of prions. This finding is concordant with studies revealed that disrupted autophagy leads to exacerbated release of α-synuclein aggregates in exosomes that increased intercellular transmission of this detrimental protein to neighboring neurons (35, 79). Moreover, a dysfunctional autophagy is a characteristic of the elderly brain, associated with release of undigested or partially digested aggregates in exosomes (80). This could be a contributing factor for the high prevalence of neurodegenerative diseases in this age group.

### CAD5 cells have less efficient autophagy and release more exosomes than N2a cells

Interestingly, our data show that PrP^Sc^ and prion-conversion activity released in exosomes isolated from ScN2a cells were miniscule compared with that released from ScCAD5 cells. One possible explanation is that the subcellular trafficking of PrP^Sc^ is different between ScN2a and CAD5 cells. It has been reported previously that PrP^Sc^ does not co-localize with flotillin in ScN2a cells, whereas CNS-derived ScGT1–7 cells show an abundance of flotillin-PrP^Sc^–rich compartments (81).

On the other hand, this was not only true for PrP^Sc^. It was also a general feature that CAD5 cells release higher amounts of exosomes than N2a cells do. The weak basal level of autophagy in CAD5 cells compared with N2a cells as shown by LC3-II signals could be one of the reasons making CAD5 cells better exosome releasers than N2a cells. However, the difference in exosome release in our two cell lines does not seem to be related to PrP^C^ level. We propose that the difference in basal autophagy and consequently in exosomal spreading of prions between CAD5 and N2a cells could be a contributing factor for the difference in their response to prion infection (CAD5 is more susceptible to infection). However, further studies on different cell lines are required to test whether the weak basal autophagy is a general feature for the central neuronal cells, which show sever neuronal loss in response to prion infection

Taken together, there is an intricate cross-talk between autophagy and exosome release that profoundly influences lateral transmission of the prions. This interplay represents a novel target for developing therapeutic approaches to fight prion and prion-like diseases.

## Experimental procedures

### Reagents and antibodies

Unless otherwise indicated, all the reagents and chemical were obtained from Sigma–Aldrich. Proteinase K (PK; 03115879001) and Pefabloc inhibitor (11286700) were from Roche. Lipofectamine 2000 (Invitrogen, 1166807), Lipofectamine LTX transfection reagents (Invitrogen, 15338100), and On Target plus mouse Atg5 (11793) siRNA Smart Pool (GE Health Care, L-064838-00-0005) were acquired from the manufacturers indicated in parentheses.

Sources of the antibodies were as follows: anti-flotillin-1 (BD Transduction Laboratories, 610820), anti-Tsg101 (Santa Cruz Biotechnology, sc-7964), HSC70 (Thermo Scientific, PA5-27337), CD63 (Santa Cruz Biotechnology, SC-15363), Alix (Cell Signaling, 2171), anti-CD9 (Abcam, ab92726), Bcl2 (Cell Signaling, D17C4), Atg-5 (clone 7C6) (NanoTools, 0262-100), nucleoporin P62 (BD Transduction Laboratories, 610497), anti-GM130 (BD Transduction Laboratories, 610822), anti-β-actin mAb (Sigma–Aldrich, A5441), anti-LC3 (clone 2G6) (NanoTools, 0260-100), anti-PrP mAb 4H11 has been previously described (52). Peroxidase-conjugated Igs were from Jackson Immunoresearch Lab (goat anti-mouse HRP, 115-035-003; and goat anti-rabbit HRP, 111-035-45).

### Maintenance of cell culture

The mouse neuroblastoma cell line N2a was obtained from ATCC (CCL-131), and they were cultured in Gibco Opti-MEM GlutaMAX medium (Gibco, 51985-34) containing 10% fetal bovine serum (Sigma, F1051), and penicillin/streptomycin in a 5% CO_2_ atmosphere. CAD5 cells are a central nervous system catecholaminergic cell line (82). The cells were subjected to single cell sub-cloning to be optimized for the prion infection (60). The cells were a generous gift from Dr. S. Mahal (Scripps Institute–Florida) and were cultured in Opti-MEM GlutaMAX medium containing 10% bovine growth serum (Hyclone, SH30541.03), and penicillin/streptomycin in a 5% CO_2_ atmosphere. The cells were infected with 10% brain homogenate of mouse-adapted scrapie strain 22L. For rapamycin treatment, the cells were treated with 500 nm of rapamycin or solvent only (DMSO) for 6 days. Firstly, rapamycin was added to complete media for 3 days; then complete medium was replaced with exosome-free medium, and rapamycin was added for another 3 days. For wortmannin treatment, the cells were cultured in complete medium for 48 h. Then complete medium was replaced with exosome-free medium, and the cells were treated with 4 nm of wortmannin for 48 h or solvent only–treated (DMSO). The cells were treated with 100 nm of bafilomycin A1 or vehicle only (DMSO) for 4 h.

### Exosome isolation

Exosomes were isolated from five confluents (15-cm plates); each plate has ∼15 million cells. For the exosome collection, the culture medium was replaced by exosome-free medium 48 h after seeding the cells into 15-cm plates. The cells were left for 2 or 3 days as indicated, and the exosomes were isolated from culture medium by differential ultracentrifugation according to the Clayton group (23) using a Beckmann Coulter Optima XE-90 ultracentrifuge. Briefly, the culture supernatant was centrifuged at 2000 × *g* for 10 min; the pellet was discarded (dead cells); the supernatant was centrifuged again at 10,000 × *g* for 30 min; the pellet was discarded (cell debris); the supernatant was ultracentrifuged at 100,000 × *g* for 70 min; then the supernatant was discarded; and the pellet contains the exosomes. The pellet was washed one time with PBS and ultracentrifuged at 100,000 × *g* for 70 min. Exosome pellet was resuspended in 100 μl of PBS and then transferred to a continuous sucrose gradient, and 10 fractions were collected after ultracentrifugation at 285,000 × *g* for 50 min.

### Knockout of Atg5 CRISPR/Cas9 in N2a cells

Expression vectors for guide RNA (sgRNA) were made targeting two exons of the Atg5 gene: exons 5 and 6. sgRNAs against exons 5 and 6 were purchased from Sigma. The following constructs were used: exon 5: target ID MM0000476061, vector U6gRNA-Cas9–2A-GFP, target sequence CCTCAACCGCATCCTTGGATGG; and exon 6, target ID MM0000476062, vector U6gRNA-Cas9–2A-RFP, target sequence GCCATCAACCGGAAACTCATGG. N2a-K21 cells were co-transfected with sgRNA and Cas9 expression vectors by Lipofectamine Plus LTX reagent according to the manufacturer's protocol. We targeted exons 5 and 6 together in double transfection. Transient GFP/RFP expression was used as transfection effectiveness control. Single cell clones were analyzed by immunoblotting for Atg5 and LC3-II and by DNA sequencing upon PCR amplification of genomic DNA.

### PK digestion and Western blotting

Immunoblot analysis was previously described (59). Briefly, confluent cells or exosome pellet was lysed in cold lysis buffer (10 mm Tris-HCl, pH 7.5, 100 mm NaCl, 10 mm EDTA, 0.5% Triton X-100, 0.5% sodium deoxycholate) for 10 min. Aliquots of lysates were incubated with PK for 30 min at 37 °C; PK was stopped by addition of proteinase inhibitors (0.5 mm Pefabloc) and directly precipitated with methanol. For samples without PK treatment proteinase inhibitors were added directly and precipitated with methanol. Precipitated proteins were resuspended in TNE buffer (50 mm Tris-HCl, pH 7.5, 150 mm NaCl, 5 mm EDTA). For the gradient, the PK digestion was carried out after the fractionation. The samples were run on 12.5% SDS-PAGE, electroblotted on Amersham Biosciences Hybond P 0.45 PVDF (Amersham Biosciences, 10600023) and analyzed in immunoblot, using the Luminata Western Chemiluminescent HRP Substrates (Millipore, WBLUF0100).

### Transmission electron microscopy

The exosome pellets were fixed with 2% paraformaldehyde (Electron Microscopy Sciences, 15700) and 2.5% glutaraldehyde (Electron Microscopy Sciences, 16220) in 0.1 m cacodylate buffer, pH 7.4, for 2 h. The fixation supernatants were discarded, and warm 1% agarose was added over the pellet. After the cooling of the agarose, the embedded pellet was cut into pieces and washed three times with same buffer. Then the pieces were postfixed in 1% osmium tetroxide (Electron Microscopy Sciences, 19100) in cacodylate buffer (Electron Microscopy Sciences, 11652) for 1 h, dehydrated through a graded series of ethanol, and embedded in Spurr resin (Electron Microscopy Sciences, 14300). Ultrathin sections were cut in a Leica EM UC7 ultramicrotome using a diamond knife and stained with 2% aqueous uranyl acetate and Reynolds's lead citrate. The sections were examined under a Hitachi H7650 transmission electron microscope at 80 kV. The images were acquired through an AMT 16000 CCD fixed on the microscope.

### XTT cell viability assay

The cell viability was assessed by TACS® XTT cell proliferation Assay (Trevigen, 4891-025-K) according to the manufacturer's instructions. Absorbance was measured at 490 nm using BioTek Synergy HT. The data were expressed as percentages of viability compared with the corresponding control.

### LDH cytotoxicity assay

The assay was conducted according to the manufacturer's protocol (Roche, 11644793001). Briefly, cell supernatant was centrifuged to remove cell debris. In a 96-well plate, 100 μl of cell supernatant and 100 μl of LDH reagent were mixed and incubated away from light for 10 min. The reaction was stopped using 50 μl of 1 n HCL. Measurement of the color intensity was done at 490 nm. At least six replicates were done for every condition.

### Primary prion infection

#### 

##### Infection using cell homogenate

Either uninfected CAD5 or persistently infected ScCAD5 (22L strain) cells were harvested from one plate (10 cm). Culture medium was removed, followed by five washes with PBS, and then cells were mechanically detached by pipetting. The cell suspension was collected and centrifuged (200 × *g*, 5 min). Supernatant was discarded, and the cells were resuspended in PBS (180 μl). The cells were centrifuged again, the supernatant was discarded, and the pellet resuspended in PBS. The cells were transferred to cryogenic vials and stored at −20 °C for 2 h, thawed, and homogenized by passing through a 25-gauge needle 50 times.

##### Infection using brain homogenate

The mouse-adapted scrapie strain 22L was propagated in C57Bl/6 mice. The mice were euthanized when they were terminally sick. To prepare brain homogenates, brain was homogenized in PBS at a final concentration of 10% (w/v) using a gentle MACS^TM^ dissociator at room temperature for 2 min, followed by centrifugation at 2,000 × *g* for 1 min, then aliquoted, and stored at −80 °C.

##### Infection using exosomes

Exosomes were collected from cell culture medium of 15 plates (10 cm) of either uninfected CAD5 or ScCAD5 (22L) cells. For primary infection, 1 × 10^5^ cells were overlaid with 50 μl of cell homogenate, brain homogenate, or exosome preparation in 450 μl of serum-free culture medium. After 5 h of incubation, 500 μl of complete culture medium was added. Medium was removed 24 h later, and the cells were washed once with PBS before fresh culture medium was added to the cells. The cells were passaged several times and subjected to PK digestion for detection of PrP^Sc^ in immunoblot.

### Real time quaking-induced conversion

#### 

##### Preparation of recombinant protein

The recombinant mouse PrP protein preparation was conducted according to Caughey group (63). Briefly, *Escherichia coli* has a pET41 vector with the mouse PrP sequence cultured in LB medium supplemented with kanamycin (0.05 mg/ml) and chloramphenicol (0.034 mg/ml), and the Overnight Express Autoinduction System 1 (Novagen, 71300) was used to induce the protein expression. The inclusion bodies were isolated from the pelleted bacteria using the Bug Buster Master Mix (Novagen, 71456) and stored at −20 °C. For purification of the recombinant PrP, the isolated inclusion bodies were dissolved into 8 m guanidine HCl in 100 mm sodium phosphate, pH 8.0, and then incubated on the rocker for 1 h at room temperature. Nickel–nitrilotriacetic acid Superflow resin beads (Qiagen, 1018401) were incubated in denaturing buffer (6 m guanidine HCl, 100 mm sodium phosphate, 10 mm Tris, pH 8.0) for 1 h at room temperature. Solubilized inclusion bodies were centrifuged at 7900 × *g* for 5 min, and the supernatant was added to the beads and incubated for 1 h with gentle rocking. The beads were then packed into a XK16 glass column (GE Healthcare Life Sciences; 28988937; length, 200 mm). Using an Amersham Biosciences AKTA Explorer FPLC running Unicorn software (version 5; GE Healthcare Life Sciences), the protein was refolded by a gradient from 100% denaturing buffer to 100% refolding buffer (100 mm sodium phosphate, 10 mm Tris, pH 8.0) over 4 h. Afterward, the column was washed for 30 min with refolding buffer. The protein was then eluted from the column using a linear gradient from 100% refolding buffer to 100% elution buffer (500 mm imidazole, 100 mm sodium phosphate, 10 mm Tris, pH 5.8). The central portions of the A280 UV peak were collected into dialysis buffer (10 mm sodium phosphate buffer, pH 5.8). The purified protein was then filtered with a 0.22-μm filter, transferred into Slide-A-Lyzer dialysis Cassette (molecular mass, 10 kDa; Thermo-Scientific, 87731), and placed in a 4-liter beaker of dialysis buffer overnight at 4 °C with continuous stirring. Following dialysis, the protein solution was filtered again with a prewashed 0.22-μm Argos syringe filter, measured using BCA protein assay (Thermo Scientific, 23227), aliquoted, and kept at −80 °C until use.

##### RT-QuIC assay

98 μl of the master mix containing a final concentration of 10 mm phosphate buffer (pH 7.4), 300 mm NaCl, 0.1 mg/ml rPrP, 10 μm ThT, and 1 mm of EDTA was loaded into each well of a black-walled 96-well plate with a clear bottom (Nunc, 165305), and reactions were seeded with 2 μl of the indicated test dilution for a final reaction volume of 100 μl. All reactions contained a final concentration of 0.002% SDS. The reactions were incubated at 42 °C and shaken every other minute at 700 rpm. Brain homogenate, cell, or exosomes lysate serial dilutions were prepared using 0.5-ml microtubes. The plates were sealed and incubated in FLUOstar Omega (BMG Labtech, Cary, NC) for 30 h with cycles of 60 s shaking and 60 s of rest throughout the incubation. ThT fluorescence measurements (450 nm excitation and 480 nm emission; bottom read) were taken every 15 min. To analyze the RT-QuIC, the data were averaged from four replicate wells and normalized to a percentage of the maximal fluorescence response of the instrument. The average values were plotted against the reaction times. The samples were scored positive if at least 50% of replicates reached a ThT fluorescence cut-off.

### Statistical analysis

Statistical analysis was performed using GraphPad InStat (GraphPad Software Inc., version 3.05; Ralf Stahlman, Purdue University) using either the unpaired two-tailed *t* test for pair-wise comparisons or the one-way analysis of variance analysis with Tukey post test for multiple comparisons. Statistical significance was expressed as follows: *ns*, not significant; *, *p* < 0.05; **, *p* < 0.01; ***, *p* < 0.001. The graphs were plotted using GraphPad Prism 7.

## Author contributions

B. A. A., D. H. A., and H. M. S. conceptualization; B. A. A., D. H. A., and H. M. S. data curation; B. A. A., D. H. A., and H. M. S. formal analysis; B. A. A., D. H. A., and H. M. S. validation; B. A. A. and D. H. A. investigation; B. A. A., D. H. A., and H. M. S. methodology; B. A. A., D. H. A., and H. M. S. writing-original draft; D. H. A. and H. M. S. writing-review and editing; H. M. S. resources; H. M. S. supervision; H. M. S. funding acquisition; H. M. S. project administration.

## Supplementary Material

Supporting Information

## Footnotes

3 The abbreviations used are:

CNS: central nervous system
TEM: transmission EM
RT-QuIC: real-time quaking-induced conversion
MVB: multivesicular body
PK: proteinase K
RFU: relative florescence unit(s)
sgRNA: guide RNA
ThT: thioflavin-T
XTT: 2,3-bis-(2-methoxy-4-nitro-5-sulfophenyl)-2*H*-tetrazolium-5-carboxanilide.
