# Supplementary material for: Autophagy regulates exosomal release of prions in neuronal cells
Source: J Biol Chem. 2018 Apr 26;293(23):8956–68. doi: 10.1074/jbc.RA117.000713 (PMC5995502; doi:10.1074/jbc.RA117.000713)
Supplement: Supporting Information [file supp_293_23_8956__index.html]

Autophagy regulates exosomal release of prions in neuronal cells — Autophagy controls exosomal release of prion — Autophagy regulates exosomal release of prions in neuronal cells — Autophagy controls exosomal release of prions — Supporting Information 

# Autophagy regulates exosomal release of prions in neuronal cells

## Supporting Information

- Supporting Information - supporting information to be published (SFig. 01 -03).
