# Supplementary material for: Autophagy regulates exosomal release of prions in neuronal cells
Source: J Biol Chem. 2018 Apr 26;293(23):8956–68. doi: 10.1074/jbc.RA117.000713 (PMC5995502; doi:10.1074/jbc.RA117.000713)
Supplement: Supporting Information [file supp_RA117.000713_133428_2_supp_126931_p7qvt9.pdf]

Autophagy regulates exosomal release of prions in neuronal cells

Basant A. Abdulrahman, Dalia H. Abdelaziz, Hermann M. Schatzl

S-1

Figure S1: Cell culture media of infected cells contains PrP^{Sc}, and GW4869 and monensin manipulate the exosomal release in ScCAD5 and ScN2a cells. (A and B) Cell culture media of either ScCAD5 or ScN2a, respectively, were subjected to differential centrifugation. The media was submitted to a first centrifugation for 10 min at 2,000 x g to remove dead cells. The supernatant was ultracentrifuged at 10,000 x g for 30 min to remove cell debris. The resulting pallet of each centrifugation step was analyzed by Western blot for its PrP content before and after PK digestion. (C and D) Western blot of exosomes of ScCAD5 and ScN2a cells, respectively. Cells were treated with 5 µM of exosomal inhibitor GW4869 (GW) or vehicle only (DMSO). Cells were cultured in complete media for 2 days. Then, complete media was replaced with exosome-free media, with or without GW. After 2 days exosomes were prepared from cell culture media. Flotillin-1 was used as exosome marker. PrP was probed with mAb 4H11. (E and F) Western blot of exosomes of ScCAD5 and ScN2a cells, respectively, treated for 2 days with 0.5 µM monensin or vehicle control (EtOH). Flotillin-1 (Flot-1) was used as exosomal marker. PrP was probed with mAb 4H11.

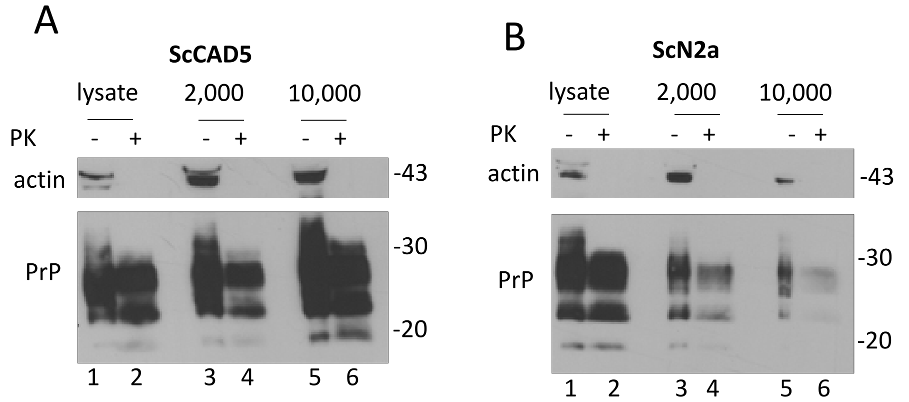

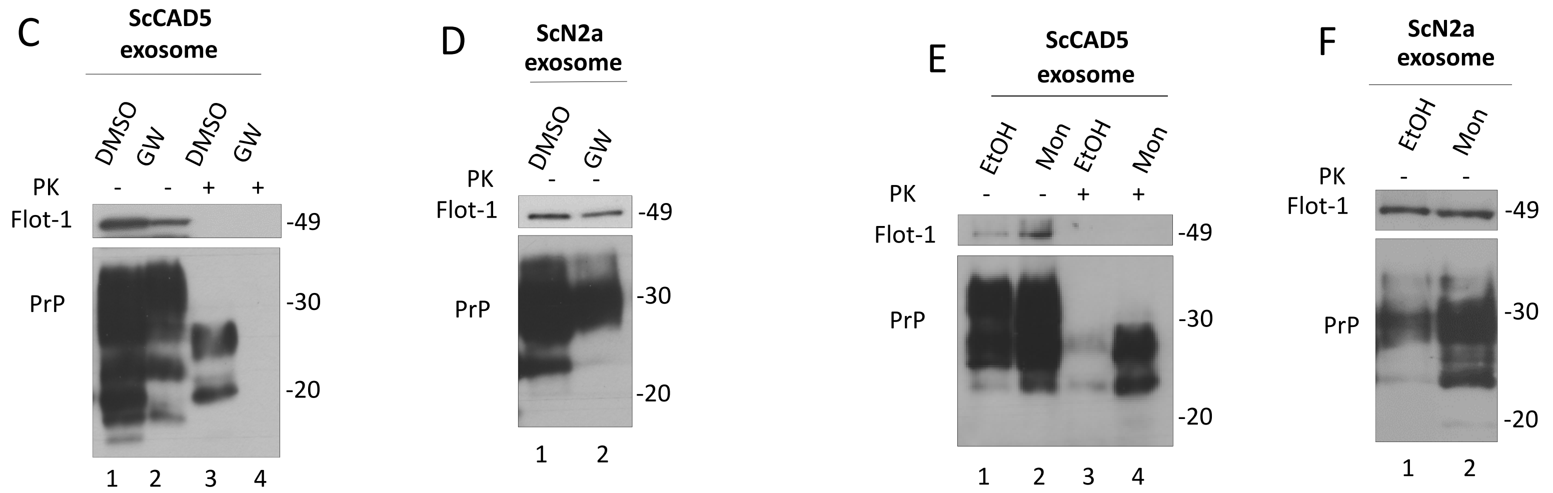

S-2

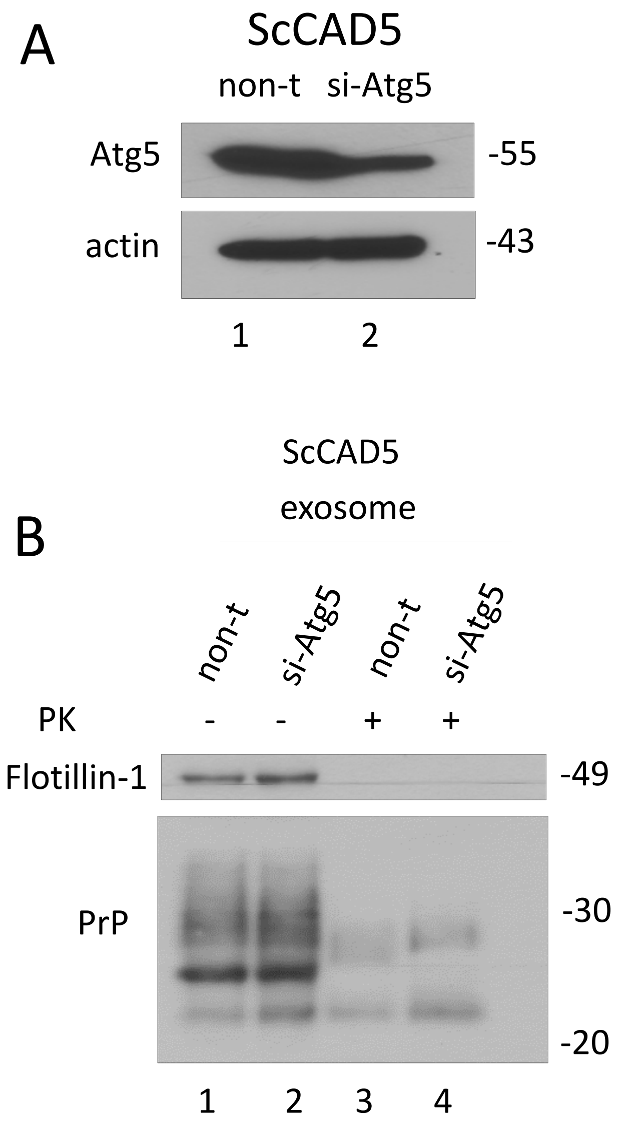

Figure S2: Autophagy knockdown using siRNA. (A) Western blot of ScCAD5 cells upon transient knockdown of Atg5 by siRNA (si-Atg5; 72 h post transfection) showing successful knockdown of Atg5. Cells treated with non-targeting siRNA (non-t) were used as control. Actin was used as a loading control. (B) Exosome isolate from Atg5-targeted and control ScCAD5 cells were analyzed in immunoblot for total PrP and PrP^{Sc} (-/+ PK, mAb 4H11). Transfection was done for three days, exosome-free media was present for two days before exosomal preparation. Flotillin-1 was used as exosomal marker.

S-3

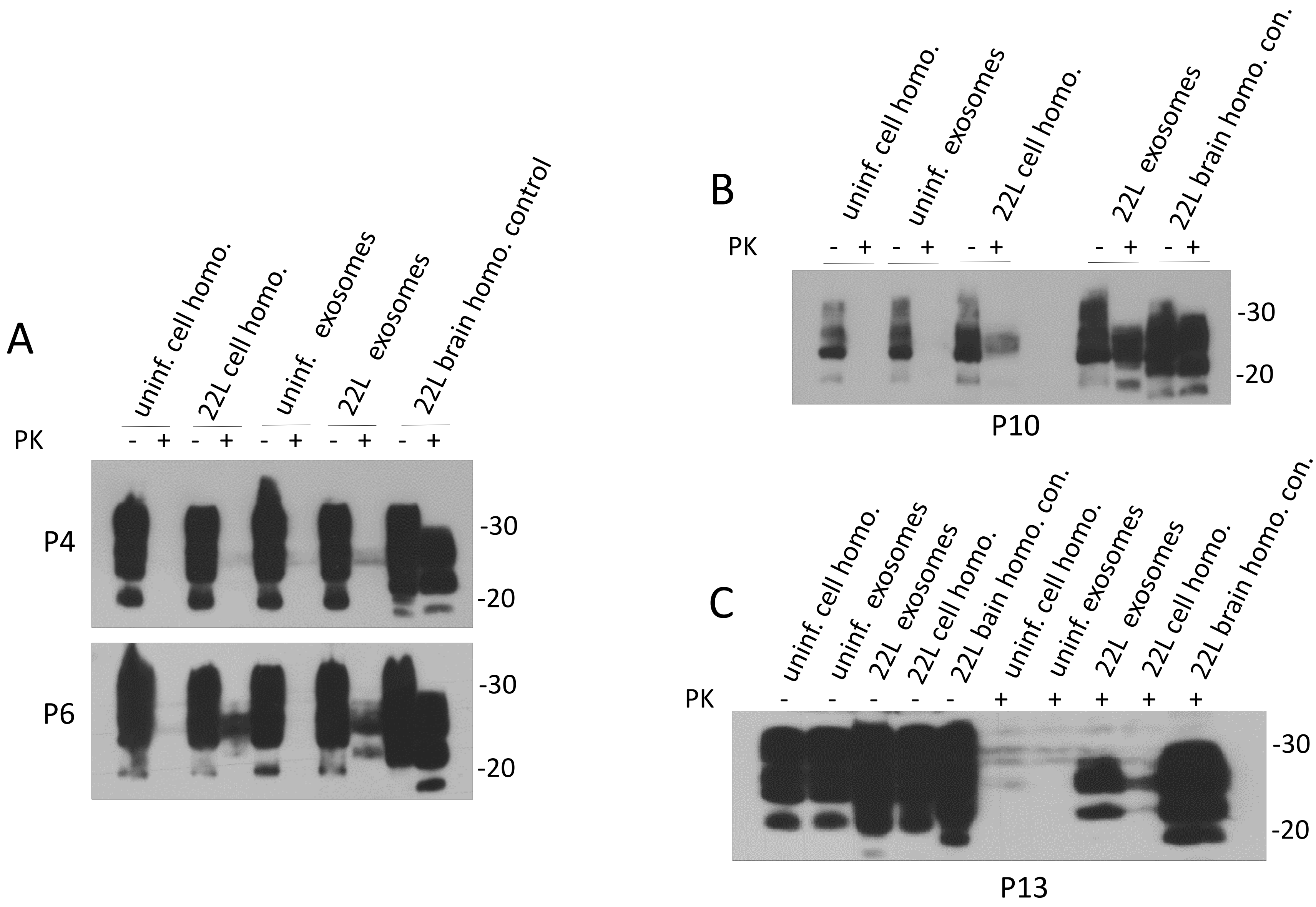

Figure S3: ScCAD5 exosomes induce prion conversion in uninfected CAD5 cells. Uninfected CAD5 cells were incubated overnight with either uninfected CAD5 cell homogenate, uninfected CAD5 exosomes, ScCAD5 cell homogenate (22L prion strain) or ScCAD5 exosomes (22L prion strain). Brain homogenate (22L prion strain) was used as immunoblot positive control. Cells were passaged several times (P4, P6, P10 and P13) and tested for prion propagation using 4H11 antibody.
